# Autoimmune Liver Diseases and Rheumatoid Arthritis—Is There an Etiopathogenic Link?

**DOI:** 10.3390/ijms25073848

**Published:** 2024-03-29

**Authors:** Ioana Ruxandra Mihai, Ciprian Rezus, Maria Alexandra Burlui, Anca Cardoneanu, Luana Andreea Macovei, Patricia Richter, Ioana Bratoiu, Elena Rezus

**Affiliations:** 1 Department of Rheumatology, Faculty of Medicine, “Grigore T. Popa” University of Medicine and Pharmacy, 700115 Iasi, Romanialuana.macovei@umfiasi.ro (L.A.M.); elena.rezus@umfiasi.ro (E.R.); 2 Clinical Rehabilitation Hospital, 700661 Iasi, Romania; 3 Department of Internal Medicine, Faculty of Medicine, “Grigore T. Popa” University of Medicine and Pharmacy, 700115 Iasi, Romania; 4 “Sfantul Spiridon” Emergency Hospital, 700111 Iasi, Romania

**Keywords:** rheumatoid arthritis, autoimmune liver diseases, primary biliary cirrhosis, autoimmune hepatitis, primary sclerosing cholangitis

## Abstract

Rheumatoid arthritis (RA) is a systemic immune-mediated disease that, in addition to the articular involvement, can have extra-articular manifestations. Even though liver damage in RA is not very common, associated autoimmune liver diseases (AILDs) may occur. The most common AILD associated with RA is primary biliary cirrhosis (PBC), followed by autoimmune hepatitis (AIH) and primary sclerosing cholangitis (PSC). There are common underlying mechanisms that play a role in the emergence of autoimmunity and inflammation in both rheumatic and autoimmune liver diseases. Genetic studies have revealed the existence of several common disease-associated genes shared between RA and AILDs, and infectious triggers, particularly those associated with recurrent or complicated urinary tract infections, are also speculated to be potential triggers for these conditions. Moreover, these diseases share common serologic patterns characterized by the presence of specific autoantibodies and hyper-gammaglobulinemia. In this study, we focus on reviewing the association between RA and AILDs regarding the prevalence and possible etiopathogenic link.

## 1. Introduction

Rheumatoid arthritis (RA) is a systemic autoimmune condition known for its symmetrical polyarticular joint involvement. It often leads to extra-articular manifestations involving the cardiovascular, pulmonary, and hematological systems [1]. Even though liver complications in RA are relatively uncommon, and liver involvement is not commonly acknowledged as a notable extra-articular manifestation of RA, irregularities in hepatic function tests have been observed in a substantial percentage of RA patients, varying from 5% to 77% [1,2,3,4]. However, the importance of these irregularities in a clinical context remains uncertain [5]. In cases of autoimmune rheumatic disorders, if there is evidence of liver damage, discerning whether it represents a hepatic manifestation of the rheumatic condition, a connected primary liver disease, or a hepatic injury resulting from the treatment of the rheumatic disorder can be challenging [6].

Liver damage associated with autoimmune rheumatic disorders typically manifests as asymptomatic abnormalities in liver function tests including increased aminotransferases (hepatocellular injury pattern), elevated alkaline phosphatase (ALP), and gamma-glutamyl transferase (GGT) with or without elevated levels of bilirubin (indicating a cholestatic pattern), and a combination of irregular liver test results (mixed picture) [6]. An increased level of ALP is the most commonly observed abnormal liver test result linked to RA and may be seen in up to 50% of patients [5,7]. High levels of ALP show a correlation with indicators of RA activity, such as C-reactive protein and erythrocyte sedimentation rate [8,9]. Approximately one-third of individuals with RA exhibit heightened levels of ALP, indicating potential liver involvement [9].

The most prevalent autoimmune liver disease (AILD) is primary biliary cirrhosis (PBC), followed by autoimmune hepatitis (AIH) and primary sclerosing cholangitis (PSC). These conditions may manifest independently or concurrently [1,10]. They are distinguished by the infiltration of lymphocytes to the liver, elevated liver enzyme levels, the production of autoantibodies, and linked HLA loci [11]. The connection between rheumatologic diseases and the liver is complex and not entirely understood. Distinguishing autoimmune liver diseases from liver disease caused by RA can be challenging under specific circumstances. This is because individuals with autoimmune hepatitis may exhibit symptoms that are not related to the liver that resemble rheumatoid pathways, while rheumatoid diseases can be linked to hypergammaglobulinemia and elevated levels of autoantibodies [12]. There is a substantial overlap in terms of epidemiology, genetics, and immunology among autoimmune rheumatologic diseases and autoimmune liver diseases [1,2,13,14,15]. However, conclusive diagnosis is not consistently attainable in clinical practice since liver histology is not routinely performed [9,16]. It is established that individuals with one immune-mediated disease are susceptible to additional autoimmune conditions, and this association has also been confirmed in patients with RA [6]. In general, RA can be identified in approximately 5% of individuals affected by diverse autoimmune diseases, encompassing AIH, PBC, and PSC [17].

The current review seeks to outline the discoveries related to the connection between AILDs and RA.

## 2. Material and Methods

To address the key question, a comprehensive review of the published literature was carried out by surveying databases such as PubMed, Google Scholar, EMBASE, and MEDLINE. This research utilized the following search terms relevant to the key question: “rheumatoid arthritis” AND “autoimmune liver diseases”, “rheumatoid arthritis” AND “autoimmune hepatitis”, “rheumatoid arthritis” AND “primary biliary cirrhosis”, “rheumatoid arthritis” AND “primary sclerosing cholangitis”, “rheumatoid arthritis” AND “liver”. Studies that examined potential connections between AILDs and RA were identified. We conducted a review of the publications of the research studies between 1980 and 2023, without taking into consideration the ones that were not in English. This review focused on frequently referenced published works characterized by rigorous research methodologies and substantive findings. Additionally, we examined relevant additional articles from the bibliographies of retrieved papers.

## 3. Results

### 3.1. Autoimmune Hepatitis

AIH is an autoimmune hepatic condition that most commonly affects women [18,19]. It is characterized by typical yet indistinct results in liver biopsy, the presence of autoantibodies, and high levels of serum aminotransferases and gamma-globulins, along with the absence of alternative factors contributing to the hepatic disorder, particularly viral hepatitis [18,19]. If left untreated, AIH can progress to cirrhosis and liver failure. Although the precise cause of AIH is not fully understood, its development is influenced by a complex interaction involving genetic, immunological, and environmental elements [19,20]. AIH exhibits considerable differences in the extent of disease severity and outcomes, presenting as a versatile disease with multiple clinical manifestations [21].

Despite being typically categorized into two types, depending on the presence of some particular autoantibodies (AIH type 1 (AIH-1)—antinuclear antibodies (ANAs) and smooth muscle antibodies (SMAs); AIH type 2 (AIH-2)—liver/kidney microsomal antibody type 1 (LKM1) and liver cytosol antibody type 1 (LC1)) the treatment approaches remain consistent irrespective of the subtype of the disease [20,22]. Histological examinations reveal periportal hepatitis characterized by lymphocytic infiltrates, plasma cells, and piecemeal necrosis. Additionally, lobular hepatitis may also be observed [23]. The clinical presentation of AIH spans a broad spectrum, ranging from no symptoms or mild symptoms to severe cases of fulminant hepatic failure. This variation may also differ among various ethnic groups [19,24]. AIH exhibits features reminiscent of PSC and PBC, with overlap reported in 10–20% and 2–8% of cases, respectively [25,26].

In addition, up to 40% of AIH patients may experience associated concurrent autoimmune diseases, potentially concealing the underlying liver disorder [27]. The occurrence of concurrent extrahepatic immune-mediated conditions, like autoimmune thyroiditis, diabetes, ulcerative colitis, and RA, is notably common. Autoimmune disorders often co-occur more frequently in women diagnosed with AIH type 1, especially when they test positive for HLA-DR4 [28]. Additionally, older individuals with AIH are observed to have a higher prevalence of simultaneous rheumatic conditions compared to younger adults [29]. Reports from various continents around the world indicate a prevalence ranging from approximately 20% to 49% among patients with AIH [20]. In Chouduri’s study, of 38 patients with AIH, 15 (39.4%) had associated autoimmune diseases, of which 2 patients presented with RA [23], while in Teufel et al.’s study, of 278 patients diagnosed with AIH, 111 (40%) were diagnosed with another autoimmune condition, of which 5 patients (1.8%) had RA [20]. Additionally, among patients diagnosed with AIH, the prevalence of RA is noted to range from 1.6% to 5.4%, as reported by Abe and Wong [30,31]. Additionally, a comprehensive population-based study was carried out in France, involving 1571 patients with arthritis who were undergoing long-term low-dose methotrexate therapy. In this study, liver biopsies from 25 RA patients with increased liver enzymes were analyzed. Among them, 13 individuals (52.5%) exhibited AIH-like lesions, characterized by inflammatory infiltrates in the portal and/or lobular regions which were abundant in plasma cells. These lesions were also associated with piecemeal necrosis or intralobular necrosis [32]. In Al-Chalabi et al.’s work, which studied the prevalence of associated immune-mediated diseases in AIH patients, RA was found in 7.8% of the cohort of individuals over 60 years old, and 4.4% within the group of patients under 60 years old [33].

### 3.2. Primary Billiary Cirrhosis

PBC is a liver condition that is histologically identified by chronic non-suppurative damage and gradual loss of intrahepatic small bile ducts, leading to fibrosis and, in the later stages, eventual cirrhosis [34,35,36]. The condition primarily affects women in their middle age [37]. Over fifty percent of individuals with PBC are diagnosed during an asymptomatic stage. The clinical picture includes asthenia, pruritus, digestive malabsorption, xanthelasma, and jaundice [35,38]. While PBC can independently result in articular manifestations, primarily arthralgia, it is not linked to synovitis or X-ray modifications [35]. Antimitochondrial antibodies (AMAs) can be identified in the serum, typically detected at titers of 1/40 or higher, and are considered the characteristic feature of the disease [34,35,36]. These antibodies demonstrate a specificity exceeding 99% and a sensitivity of approximately 92% [39,40]. To diagnose PBC, the following criteria are required: (1) biochemical indicators of cholestasis, indicated by increased ALP and GGT, (2) the existence of disease-specific antimitochondrial antibodies, and (3) histological characteristics consistent with PBC [41]. Currently, a diagnosis of PBC necessitates fulfilling two out of the three criteria [41]. Furthermore, it is typical to observe increased levels of immunoglobulin M (IgM) [41,42]. Liver biopsies typically reveal signs of persistent non-suppurative inflammation, cholangitis, and fibrosis. The classification by Ludwig and Scheuer outlines four stages: portal damage, periportal damage, septal damage, and cirrhosis [1,43].

Sjögren’s syndrome and systemic sclerosis, along with other extra-hepatic autoimmune manifestations such as autoimmune thyroiditis or RA, are often concurrently present with PBC [37,42,44]. PBC and RA are chronic medical conditions where autoimmune features are predominant, despite the indefinite etiology. Both are multisystemic diseases, exhibiting a wide range of manifestations beyond their primary target organ. It was firmly confirmed that individuals with PBC could experience musculoskeletal symptoms, and, conversely, individuals with RA may demonstrate signs of impaired liver function [45]. The proinflammatory cytokine tumor necrosis factor alpha (TNFa) was demonstrated to have a significant pathogenetic role in both PBC and RA [46]. PBC is the most common coexisting AILD in RA, with a prevalence of from 3.8% to 6.3% [6,15,30,47,48], while the occurrence of RA in PBC has been reported to be from 1.8% to 13% [49,50,51]. Pak suggested that patients diagnosed with RA face an elevated risk of developing PBC compared to the population at large and that, consequently, when patients with RA exhibit irregularities in liver function tests, particularly in the absence of alternative causative factors, a comprehensive investigation for PBC is warranted [36]. Reports indicate that the majority of individuals with both RA and PBC typically experience the onset of RA several years before the development of PBC [36]. In Siegel et al.’s study, the majority of the patients (n = 17) received the diagnosis of RA first, with an average lead time of 11.8 years before the diagnosis of PBC (range 2–27). For those who were diagnosed with PBC initially (n = 5), the diagnosis of RA occurred an average of 5 years later (range 2–14). In two cases, the patients were simultaneously diagnosed with both diseases. The cohort exhibited elevated transaminases, ALP, and bilirubin levels, and 13 patients were classified to be in stages 1 or 2 of PBC. According to the findings, they recommended testing for AMA in all RA individuals with irregularities in their liver function tests [45]. Bakula suggested that RA and PBC coexist in up to 6% of cases [52], while Radovanović-Dinić showed that PBC occurred in up to 10% of patients [6]. In a study by Sherlock and Scheuer, it was observed that 5% of a cohort comprising 100 PBC patients had concurrent RA, and rheumatoid factor (RF) was positive in around half of the PBC patients [53]. In Prince et al.’s study, which identified two sets of PBC cases, the odds ratio for RA in the epidemiological group was 1.52 compared to 1.21 in the support group [54]. Gershwin et al. also conducted a study that revealed that 10% of 1032 PBC cases were also associated with RA, while, in the control group (1041 patients), RA was reported in 8% of the cases. In 26% of the first-degree relatives (FDRs) of PBC patients RA was documented, compared to in 22% of the family members of the control group [55]. In Corpechot’s study, RA was identified in 3% of PBC cases (out of 222 patients) and in 1% of the controls, but this difference was not deemed statistically significant [56]. Similarly, 2% of the FDRs of PBC cases and 1% of the FDRs of the controls reported RA, and this also did not show statistical significance [56]. Higher rates of RA were observed in PBC cohorts and among female FDRs, suggesting a potential common underlying factor for both conditions in certain instances. Regarding autoantibodies, AMA was found in 0.9% of 997 RA patients in a study by Invernizzi [40], while, in Datta’s study, 18% of the RA patients tested positive for AMA [57].

In the literature, isolated cases of associations between RA and PBC have been described, as shown in the table below (Table 1). In most of the reported cases, PBC appeared after RA, AMA was positive, the level of hepatic enzymes was increased, and the evolution of the patients was favorable under treatment with ursodeoxycholic acid.

### 3.3. Primary Sclerosing Cholangitis

PSC is a persistent liver condition marked by cholestasis, involving injury to intrahepatic or extrahepatic (or both) bile ducts [38,66]. The clinical manifestations mirror the sequential progression of bile duct injury and fibrosis, resulting in stricturing, cholestasis, and the development of biliary cirrhosis accompanied by advancing hepatic dysfunction [66]. PSC is less common than other AILDs and, due to its close association with inflammatory bowel disease (IBD), is most accurately perceived as a hepatobiliary manifestation of IBD [67]. The close association with IBDs is a characteristic feature of the condition, impacting approximately two-thirds of the patients [68]. Patients may exhibit signs of cholestasis, characterized by elevated levels of ALP and GGT. As the disease progresses, symptoms such as fatigue, pruritus, and pain in the right upper quadrant may develop. The cholestatic itch can occur independently or alongside jaundice. In some cases, the presentation may involve variceal bleeding, ascites, or encephalopathy, marking the progression of occult PSC to end-stage liver disease [68]. Notably, features like ascites and encephalopathy become less prominent than in hepatitic diseases until the late stages of the disease [68]. To diagnose PSC, the gold standard methods are magnetic resonance cholangio-pancreatography or endoscopic retrograde cholangio-pancreatography [38]. Although some autoantibodies, such as perinuclear anti-neutrophil cytoplasmic antibodies (p-ANCAs), anti-nuclear antibodies (ANAs), and smooth muscle antibodies (SMAs), are commonly associated with PSC, standard antibody testing is not deemed necessary to confirm the diagnosis of PSC. Liver biopsy is only recommended when cholangiographic findings are abnormal [38].

As for the association of PSC and RA, there was a 1.2% and a 3.4% prevalence of RA in two large-scale PSC cohorts [69,70]. Gow described four cases of association between RA and PSC, as shown in the table below [71] (Table 2). In three of the four cases, there was a rapid progression of PSC to cirrhosis. The combination of RA and PSC may serve as a clinical indicator for patients at a heightened risk of progressing to cirrhosis, warranting close observation [6]. PSC may be underdiagnosed since cholangio-pancreatography is not routinely performed and mild cholestatic liver function irregularities are frequently observed in patients with RA. Hence, it is crucial to consider PSC as a differential diagnosis in patients with RA and changes in their liver function parameters [71].

## 4. Discussion

The frequent observation of concurrent manifestations of diverse autoimmune diseases has been extensively documented [20]. There are common underlying mechanisms contributing to the emergence of autoimmunity and inflammation in both rheumatic and autoimmune liver diseases. The occurrence of overlapping diseases relies primarily on genetic factors, with shared susceptible loci widely present in both disorders [67]. Moreover, these diseases share similar serological profiles characterized by the existence of specific autoantibodies and hyper-gammaglobulinemia [3,72].

Regarding epidemiology, RA has an incidence of from 0.5% to 1%, with an apparent decline from north to south and from urban to rural areas [73,74]. The prevalence of AIH has been documented as 1 per 200,000 in the US overall population, and 20 per 100,000 in female patients above the age of 14 in Spain [75]. Regarding the geographical region, the annual prevalence varies from 4.0 to 24.5 per 100,000 [76]. PBC is estimated to affect 0.02% of women and 0.002% of men [64]. Population-based epidemiological research across Europe, North America, Asia, and Australia has indicated an incidence rate for PBC ranging from 0.9 to 5.8 cases per 100,000 people per year [38,77]. PSC is considered an infrequent disease, with its incidence varying geographically, but escalating to 1.3 per 100,000 people per year in Northern Europe. The prevalence of PSC exhibits variability as well, with some studies reporting rates as high as 16.2 cases per 100,000 people [38].

**Figure 1:**
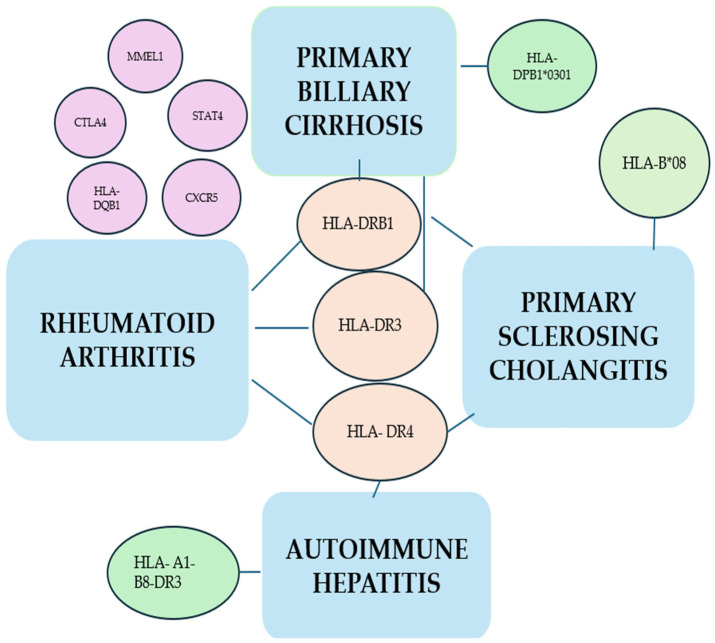
The genetic association between RA and AILDs.

Modern genetic technologies have advanced our understanding of the pathogenesis of autoimmune diseases. In RA, HLA, particularly HLA-DRB1, continues to exert a significant influence, strongly suggesting a pivotal role for (self) peptide binding in the pathogenesis of the disease [74,78]. Alleles associated with the disease have common amino acid sequences in the peptide-binding groove, known as the ‘shared epitope’ [79]. The HLA haplotypes encompassing most alleles of the DRB1*01, *04, and *10 groups include shared epitope alleles. HLA DRB*14:02 has also been identified as a significant contributor, particularly in American populations. Conversely, HLA-DRB1*13 alleles were observed to provide strong protection against RA [80,81]. The ‘shared motif hypothesis’ is a prevalent theory in numerous autoimmune diseases and has been evoked to elucidate the pathogenesis of both autoimmune hepatitis and RA [82]. The genetic link between RA and AILDs is shown in the figure below (Figure 1).

The pathogenesis of AIH remains unclear, but there is evidence suggesting an immunogenetic basis for the condition. This is evident in its well-established association, especially among northern European Caucasoids, with the inheritance of the extended HLA haplotype A1-B8-DR3 [28,83,84]. Particularly, the DR3 and DR4 allotypes within this haplotype function as autonomous risk factors for AIH, being linked with distinct clinical expressions of the disease, but also being associated with other autoimmune diseases [83,84,85]. Young age at presentation and a severe form of AIH are usually associated with DR3, whereas DR4 is more frequent among older patients, with a milder form of AIH in general [83]. In the context of type 1 AIH, an association was found with the DRB1*0405 and DRB1*0404 alleles in Argentine, Japanese, and Mestizo Mexican patients [28]. As for type 2 AIH, HLA DR7 was shown to have a high prevalence in German and Brazilian patients [86,87].

The occurrence of PBC appears to be associated with intricate interactions between genetic predisposition and environmental triggers [38]. Some North American and European research papers have demonstrated a strong connection between HLA alleles and PBC [88]. In particular, susceptibility to the disorder is linked with DRB1*08, DR3, DPB1*0301, and DRB1*08-DQA1*0401-DQB1*04, while protection has been reported to be associated with DRB1*11 and DRB1*13 [40,88]. Recent molecular investigations have indicated that PBC shares certain risk alleles with other immune-mediated diseases. These risk alleles appear to be present in genes related to immune function, potentially influencing various immune pathways. However, the specific mechanisms by which these alleles affect the phenotype are not yet fully understood [38,88,89]. Genetic investigations were carried out in an attempt to discover a connection between RA and PBC, revealing some common genes, such as histocompatibility complex, class II, DQ beta 1 (HLA-DQB1), membrane metallo-endopeptidase-like 1 (MMEL1), interferon regulatory factor 5 (IRF5), cytotoxic T-lymphocyte-associated protein 4 (CTLA4), signal transducer and activator of transcription 4 (STAT4), and CXC chemokine receptor 5 (CXCR5). This shared genetic trait predisposes individuals with RA to the development of PBC, indicating a genetic link between the two conditions [11,36,37].

The pathogenesis of PSC is still uncertain, but it appears that both genetic and environmental factors play a role in initiating the disease [68]. Numerous genes have been investigated in the context of PSC, and a strong association with human leukocyte antigen HLA class I, II, and III regions (i.e., HLA-B*08 and HLA-DRB1 alleles, and a locus near NOTCH4, respectively) has been observed [90]. In PSC, HLA DR4 is less frequently observed than in the general population, yet it has been noted that HLA DR4 may serve as an indicator of fast disease evolution [91]. Additionally, in Gow’s study, the three patients expressing HLA DR4 experienced a rapid progression to end-stage liver disease, 14–48 months after the diagnosis of PSC, whereas, in the patient with the classical PSC HLA haplotype B8-DR3, the disease had not advanced [71].

In AILDs, it is imperative to detect concurrent rheumatological diseases early on through autoantibody screening, as the coexistence of these conditions may impact their natural course and disease prognosis [11]. Up to 27% of patients with AILDs have elevated levels of RF [16,67]. Antibodies to cyclic citrullinated peptides (ACPAs) are serological markers that can be found in both rheumatic diseases and autoimmune hepatitis [92]. In one study, ACPAs were detected in 9% of patients with autoimmune hepatitis, and their presence was not dependent on coexisting RA [93]. Moreover, the strong association between ACPAs and erosive arthritis suggests that these antibodies may play a pathogenic role and could serve as indicators of individuals with liver disease who are at risk of developing inflammatory joint disease. Individuals with autoimmune hepatitis and ACPAs also exhibit a higher incidence of histological cirrhosis at presentation compared to those lacking these antibodies, and they face a higher mortality rate from hepatic failure [93]. In Koga’s study, the levels of RF and ACPAs were assessed in the sera from individuals with HCV infection (n = 45), PBC (n = 73), AIH (n = 55), and RA (n = 48), and from the sera of healthy subjects (n = 23). Among the PBC patients, two (2.7%) had ACPAs, while, in the AIH group, six patients (10.5%) were positive for ACPAs [94]. The seropositivity for ACPAs in these patients was linked to a high frequency of RA association. Moreover, considering the fact that RA is typically diagnosed before PBC in patients with overlapping disease, it is recommended to screen for AMA in individuals with RA and elevated cholestatic liver enzymes [45].

Environmental triggers, including infectious agents, may also be engaged in the induction of immune-mediated diseases. RA has traditionally been linked to potential infectious triggers, such as Proteus, *E. coli*, and Epstein–Barr virus (EBV), often through molecular mimicry models [74]. Additionally, numerous studies have documented the connection between AIH and other viral infections like hepatitis A, EBV, and cytomegalovirus [95]. Moreover, presuming the existence of a shared genetic link between RA and PBC patients, it has been proposed that common infectious triggers may play a role in the induction of both diseases in certain individuals. *E. coli* stands out as one of the extensively investigated infectious triggers of PBC. This interest arises from the notable occurrence of recurrent urinary tract infections (rUTIs) in individuals diagnosed with PBC, with *E. coli* being the most frequently found bacteria in these infections [11,37]. *E. coli* infection has also been linked to RA, and, in RF-positive patients, anti-*E. coli* IgM has been found to be elevated [96]. Molecular mimicry and cross-reactivity between self and bacterial antigens are thought to contribute to the induction of PBC [37].

It is important to note that anti-TNF therapy has the potential to trigger the production of autoantibodies, including ANAs and ASMAs [97]. The utilization of TNF antagonists has been associated with the occurrence of AIH in RA, Infliximab being the most frequently implicated agent [11]. Anti-TNF therapy-induced AIH exhibits a female predominance, a latency period of 3–14 months between the initiation of therapy and the occurrence of AIH, and shows improvement upon discontinuation of the medication along with corticosteroid use [11,97,98]. In clinical practice, it is difficult to differentiate between autoimmune hepatitis and drug-induced hepatitis, as the symptoms, serological markers, and histological findings are often indistinguishable. Typically, individuals experiencing liver injury induced by anti-TNF therapy do not exhibit relapses after resolution, regardless of whether they receive immunosuppressive therapy. Additionally, ANAs disappear following steroid therapy [99].

## 5. Conclusions

In conclusion, although the association of AILDs and RA is uncommon, it is essential for the medical professional to take AIH, PBC, or PSC into consideration in patients with RA who have irregularities in their hepatic function tests after excluding other causes, for example, hepatotoxicity induced by antirheumatic drugs, hepatitis, or other liver abnormalities. RA is noted especially in PBC patients, and PBC can be observed in individuals diagnosed with RA, however not at a substantially elevated level. Recent genetic studies have revealed the existence of some genes that can be found in both RA and PBC. This finding suggests that a subset of patients may be susceptible to both disorders. While epigenetic alterations have been extensively studied in RA, similar investigations are warranted in the context of PBC. Additionally, the role of infectious agents, particularly those associated with recurrent or complicated urinary tract infections, is speculated to be a potential trigger for both conditions, highlighting the need for further research in this area. Reaching an accurate diagnosis can have a significant effect on the outcome and quality of life of patients, and on the appropriate care of such patients. To establish the connection between these conditions, additional research is required. The cooperation between hepatologists and rheumatologists has the potential to result in significant advancements in managing this complex scenario.

## Figures and Tables

**Table 1:** Association between RA and PBC.

Study	Sex, Age	Associated Diseases	Hepatic Enzymes	Antibodies	Liver Biopsy	Treatment	Outcome
Nakano, 1992 [58]	F, 46 years	RA—diagnosed 6 years before PBC	↑ ALP, GGT	ANA + AMA −	Chronic non-suppurative destructive cholangitis	Not mentioned	AMA—always negative. ALP and bilirubin levels remained constant during the following 2 years.
Liu, 2007 [59]	F, 62 years	RA—diagnosed 2 years before PBC	ALP 767 IU/L GGT 172 IU/L ALT 68 IU/L AST 104 IU/L	AMA M2 1/320 ANA 1/640 RF, ACPA +	Stage II histology	Ursodeoxycholic acid 10 mg/kg/day	Improved the pruritus and biological hepatic abnormalities.
Liu, 2007 [59]	M, 41 years	RA—diagnosed 1 year before PBC	ALP 598 IU/L GGT 562 IU/L ALT 25 IU/L AST 98 IU/L	ANA 1/320 AMA 1/640 RF, ACPA +	Stage IV histology signs of non-suppurative cholangitis with fibrosis or cirrhosis	Ursodeoxycholic acid (10 mg/kg per day) methotrexate (15 mg per week)	Methotrexate and ursodeoxycholic acid reduced the symptoms and hepatic tests’ values.
Caramella, 2007 [35]	F, 62 years	RA—a few months before PBC	ALP 234 IU/L GGT 76 IU/L AST 25 IU/L ALT 32 IU/L RF +	AMA M2 1/1000	Stage 1 histology	Ursodeoxycholic acid 12 mg/kg/day articular injections of corticosteroid and methotrexate (15 mg per week)	PBC remained asymptomatic without clinical or biological symptoms, despite the persistence of AMA. RA was controlled. Methotrexate was well accepted and liver tests remained within the typical range.
Caramella, 2007 [35]	F, 54 years	RA—diagnosed 2 years after PBC	ALP 135 IU/L GGT 89 IU/L	AMA M2 + ANA, ANCA, LKM1 −	Non-suppurative cholangitis without fibrosis or cirrhosis	Ursodeoxycholic acid 13mg/kg/day hydroxychloroquine sulphate, sulfasalazine and prednisone (8 mg per day) then switch to methotrexate	PBC has remained asymptomatic with normal hepatic tests. RA was controlled with methotrexate treatment.
Ogata, 2009 [60]	F, 54 years	RA—diagnosed 2 months after PBC	ALP 517 IU/L GGT 60 IU/L	AMA −	Confirmed PBC	Ursodeoxycholic acid 300 mg/day etanercept 50 mg/week	Disease activity was significantly improved. Improvement in liver function 6 months after the initiation of etanercept.
Polido-Pereira, 2011 [61]	F, 50 years	RA—diagnosed 18 years before PBC	ALP 403 IU/L GGT 268 IU/L	AMA 1/640 ANA 1/160	Stage III	Ursodiol therapy Rituximab 1000 mg 2 weeks apart	PBC improved. RA remained active.
Kubo, 2011 [62]	F, 51 years old	RA diagnosed before PBC (period of time not mentioned)	ALP 939 IU/L GGT 91 IU/L	AMA +	Nonsuppressive destructive cholangitis characterized by mononuclear inflammatory cells surrounding a small bile duct and classified into stage I according to Ludwig’s classification	Ursodeoxycholic acid 600 mg/day methotrexate 6 mg/week etnaercept 50 mg/week	The RA disease activity was significantly improved by etanercept. Her liver function, including serum levels of ALP and GGT, was also immediately and markedly improved.
Lazrak, 2013 [63]	F, 60 years	RA—diagnosed 1 year after PBC	ALP 4× NV GGT 3× NV	AMA-M2 1/640	Nonsuppurative cholangitis without fibrosis or cirrhosis and	Ursodeoxycholic acid 600 mg/day methotrexate 7.5 mg/week rituximab two doses of 1000 mg separated by two weeks	Good efficiency in her arthritis after five months of follow-up but her abnormal liver function tests persisted.
Bekki, 2015 [64]	M, 71 years	RA—diagnosed 1 year before PBC	AST 167 IU/L ALT 435 IU/L ALP 2539 IU/L GGT 590 IU/L	ANA × 40 AMA × 20	Marked inflammatory cell infiltration surrounding and destroying the interlobular bile ducts in the portal area	Ursodeoxycholic acid 600 mg/day	Patient’s clinical findings and biological data showed improvement. A second biopsy after 445 days of ursodeoxycholic acid treatment demonstrated significant improvement of inflammation within the portal area.
Dimipolou, 2015 [34]	F, 61 years	RA—diagnosed 3 years before PBC Hashimoto thyroiditis Osteoporosis	PA 440 IU/L GGT 240 IU/L	ANA 1:2500 AMA 1:72 RF 105 U/L	Stage III (expansion of most of the portal tracts, inflammatory infiltrate, granulomatous destruction of the bile ducts, fibrous septa and bridging necrosis)	Ursodeoxycholic acid 13 mg/kg/day hydroxychloroquine 200 mg twice daily and methylprednisolone 16 mg/day—tapered then azathioprine 100 mg/day, while hydroxychloroquine was discontinued then methotrexate	After a year, liver function was improved, but arthritis remained poorly controlled—switch to treatment with infliximab 3 mg/kg (at weeks 0.2 and 6 and then every 8 weeks) led to considerable RA improvement on both clinical and biochemical grounds within 4 months without any further deterioration of ALP levels.
Sargin, 2016 [65]	F, 72 years	RA—diagnosed 3 years after PBC	ALP 302 IU/L GGT 86 IU/L RF, ACPA, ANA −	AMA 1/40	Nonsuppurative cholangitis and interlobular bile duct destruction	Ursodeoxycholic acid rituximab cycles with two infusions every six months	Good response.
Pak, 2017 [36]	F, 56 years	RA—11 years before PBC	AST 54 IU/L ALT 49 IU/L	AMA 1/640	Increased collagen in portal areas with portal widening mild piecemeal necrosis and a mixed inflammatory infiltrate	Ursodeoxycholic acid 250 mg twice daily	While clinically asymptomatic over six months, the patients’ liver function tests remained elevated (AST 36 U/L, ALT 43 U/L). Uptitration of ursodeoxycholic acid to 500 mg twice daily reduced AST and ALT to normal.

F = female, M = male, RA = rheumatoid arthritis, PBC = primary biliary cirrhosis, ALP = alkaline phosphatase, GGT = gamma-glutamyl transferase, AST = aspartate aminotransferase, ALT = alanine transaminase ANA = antinuclear antibodies, AMA = anti-mitochondrial antibody, ACPA = anti-citrullinated protein antibodies, LKM1 = anti liver-kidney microsomal antibodies type 1, and RF = rheumatoid factor.

**Table 2:** Association between RA and PSC.

Sex, Age	Associated Diseases	Hepatic Enzymes	Liver Biopsy	ERCP	Treatment	Outcome
M, 23 years	RA—diagnosed 4 years before PSC Pan-ulcerative colitis—diagnosed 3 years before PSC	AST 100 IU/L ALP 1956 IU/L GGT 249 IU/L ANCA, RF +	Modifications of PSC with notable fibrosis	Intra- and extra-hepatic sclerosing cholangitis	High dose ursodeoxycholic acid (25 mg/kg per day) hydroxychloroquine	Distal common bile duct stricture—biliary stent the bilirubin remained elevated clinical evidence of portal hypertension. Emergency colectomy for toxic megacolon liver transplant.
F, 67 years	RA—diagnosed 20 years before PSC	AST 95IU/L ALP 3910 IU/L GGT 386 IU/L ANCA, RF −	Modifications of sclerosing cholangitis with minor fibrosis	Intrahepatic sclerosing cholangitis	High dose ursodeoxycholic acid	Remained well without symptoms or signs of liver disease.
M, 55 years	UC—diagnosed 12 years before PSC RA—diagnosed 11 years before PSC	AST 68 IU/L ALP 2050 IU/L GGT 982 IU/L ANCA- RF +	PSC with significant fibrosis	PSC involving the intra-hepatic ducts	Ursodeoxycholic acid	Gradual worsening of liver function. Developed recurrent cholangitis. Liver transplant showed proof of recurrent PSC in the transplanted liver.
F, 61 years	RA—diagnosed 20 years before PSC UC—diagnosed 1 year before PSC	AST 46 IU/L ALP 452 IU/L GGT 122IU/L ANCA- RF +	Extensive hepatic fibrosis	Narrowing and irregularity of the intra-hepatic bile ducts	Ursodeoxycholic acid sulphasalazine	Cirrhosis—stable.

F = female, M = male, RA = rheumatoid arthritis, PSC = primary sclerosing cholangitis, UC = ulcerative colitis, ALP = alkaline phosphatase, GGT = gamma-glutamyl transferase, AST = aspartate aminotransferase, RF = rheumatoid factor, and ANCA = anti-neutrophil cytoplasmic antibodies.

## Data Availability

The data presented in this study are openly available in its references.
